# Supplementary material for: The membrane curvature-inducing REEP1-4 proteins generate an ER-derived vesicular compartment
Source: Nat Commun. 2024 Oct 5;15:8655. doi: 10.1038/s41467-024-52901-6 (PMC11455953; doi:10.1038/s41467-024-52901-6)
Supplement: Supplementary file 1 — Supplemental Information [file 41467_2024_52901_MOESM1_ESM.pdf]

Supplementary Information

for

Shibata et al, "The membrane curvature-inducing REEP1-4 proteins generate an ER-derived vesicular compartment"

Supplementary Figures 1-11

Supplementary Table 1

Supplementary Figure 1

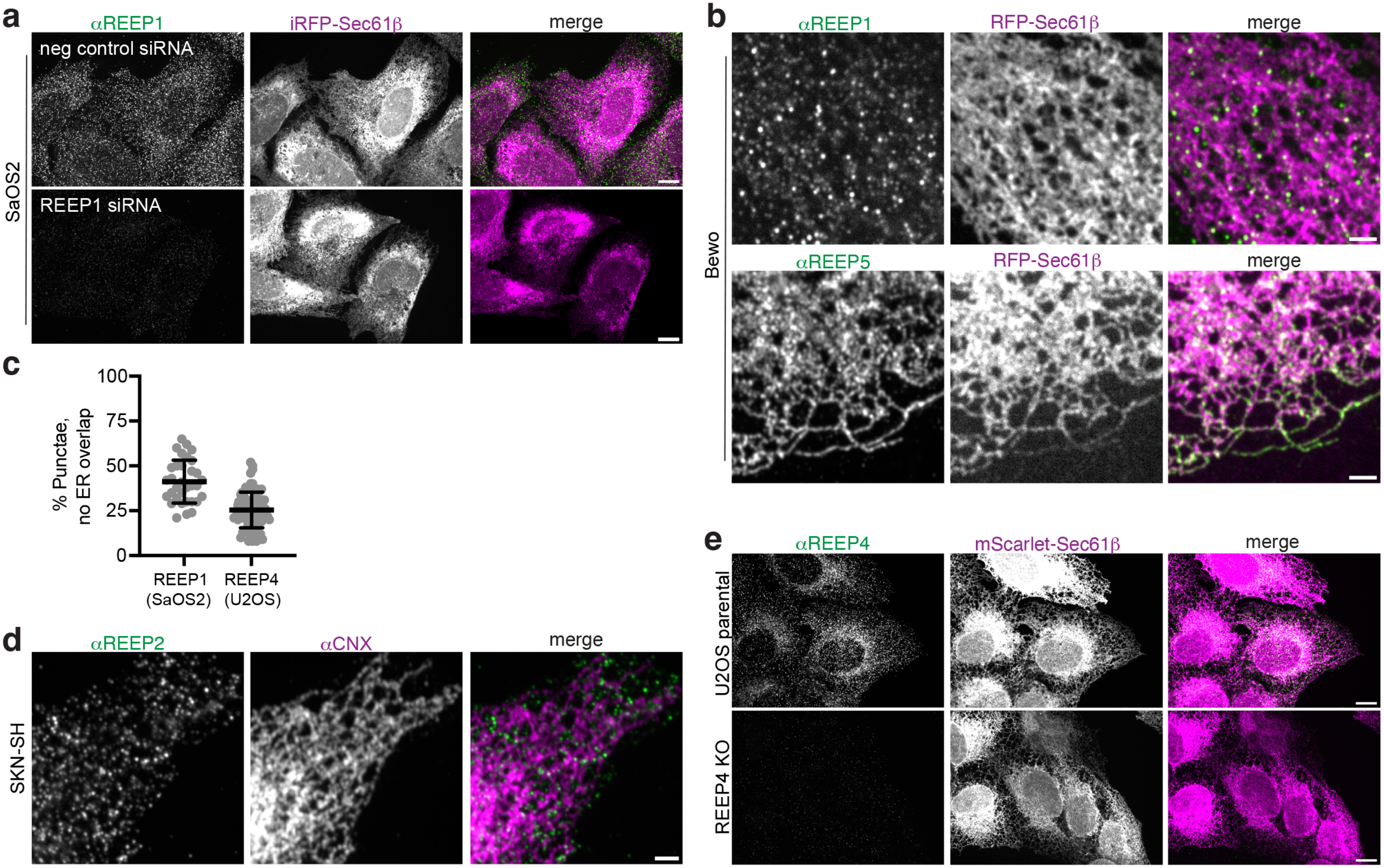

Supplementary Figure 1. Endogenous REEP1-4 proteins localize to punctae.

a, The specificity of REEP1 antibodies was tested in human SaOS2 cells stably expressing the ER marker Sec61β fused to the near-infrared fluorescent protein (iRFP-Sec61β). Cells were treated either with non-targeting siRNA oligonucleotides (negative control, top row) or with those targeting REEP1 (bottom row) and analyzed by indirect immunofluorescence with anti-REEP1 (αREEP1) antibodies. αREEP1 samples were imaged and displayed using identical settings between the two RNAi treatments. Right panels show overlays of αREEP1 (green) and iRFP-Sec61β (magenta). Scale bars, 10 μm.

b, Endogenous REEP1 (αREEP1, top row) and REEP5 (αREEP5, bottom row) localizations were analyzed by indirect immunofluorescence and confocal fluorescence microscopy in Bewo cells transfected with the ER marker construct RFP-Sec61β. Right panels, overlays between αREEP1 or αREEP5 (green) and RFP-Sec61β (magenta). Scale bars, 2 μm.

c, Quantification of endogenous REEP1 or REEP4 punctae in SaOS2 or U2OS cells, respectively, that did not overlap with bulk ER membranes marked by stably expressed iRFP-Sec61β. Each data point represents analysis from one cell. Shown are means and standard deviations. REEP1, n, 33 cells; REEP4, n, 84 cells. Exact values are listed in Source Data.

d, Endogenous REEP2 localization was determined by immunostaining with αREEP2 antibodies, compared to immuno-localization of the ER marker Calnexin (αCNX) in neuronal SKN-SH cells. Right panel, overlay between αREEP2 and αCNX (magenta). Scale bar, 2 μm.

e, The specificity of REEP4 antibodies was tested in U2OS cells stably expressing mScarlet-Sec61β by comparing αREEP4 immunostaining between parental (top row) and REEP4 CRISPR knockout cells (bottom row). αREEP4 images were taken and displayed using identical settings between the two cell lines. Right panels, overlays of αREEP4 and mScarlet-Sec61β (magenta). Scale bars, 10 μm.

Supplementary Figure 2

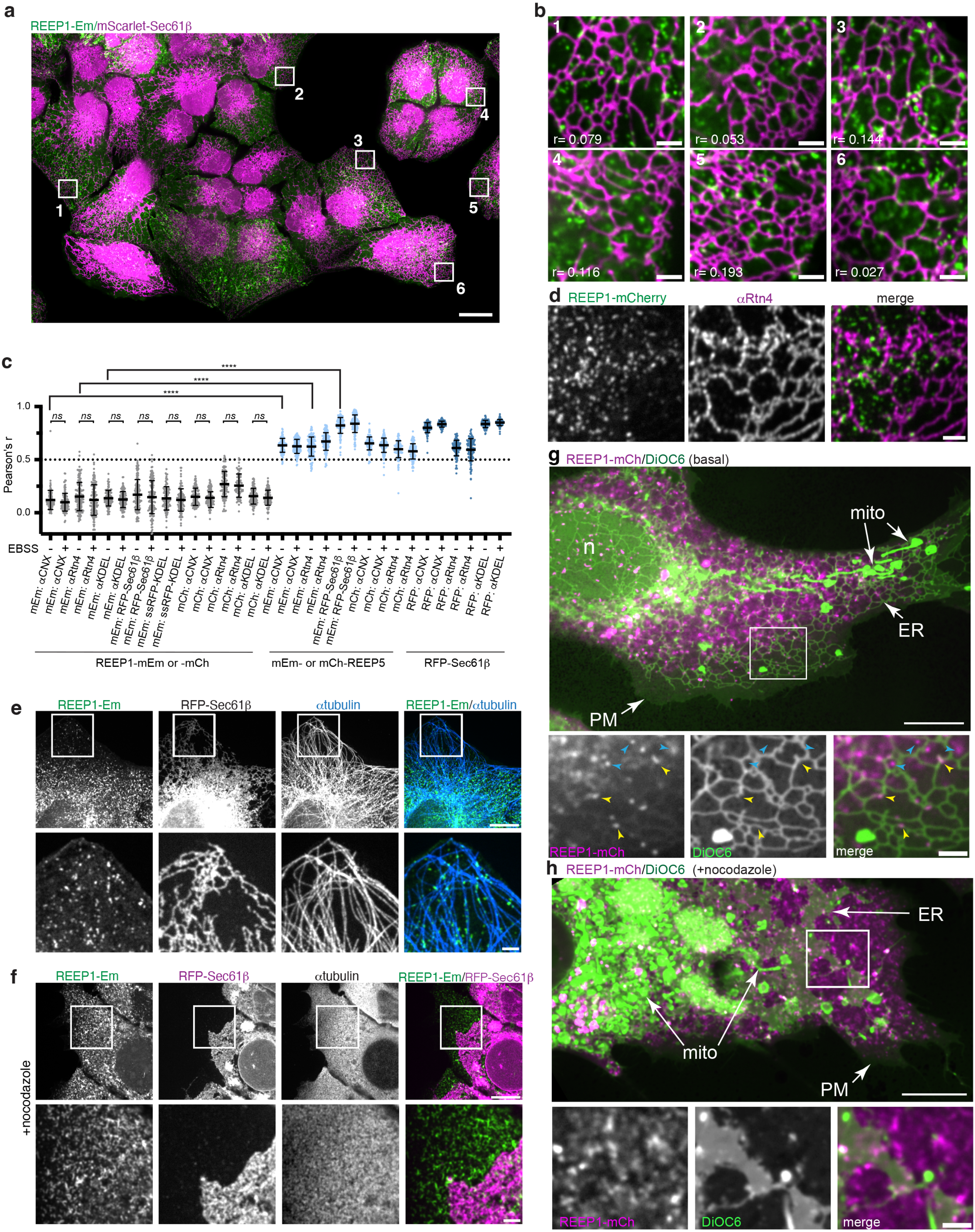

Supplementary Figure 2. REEP1 localizes to punctae independent of the bulk ER.

a, Live U2OS cells stably co-expressing REEP1 fused to mEmerald (-mEm) and the ER marker mScarlet-Sec61β were imaged by confocal fluorescence microscopy with a 100X 1.44/NA objective. Shown is a merged image (REEP1-mEm, green; mScarlet-Sec61β, magenta) of a montage stitching 3x3 fields of view. Note that REEP1-mEm localize to punctae while the ER is an intact, reticulated network in all cells. Scale bar, 20 μm.

b, Magnifications of the numbered boxed regions in a. r, Pearson's correlation coefficients calculated between the mEm and mScarlet signals. Scale bars, 2 μm.

c, Pearson's correlation coefficients comparing REEP1 localization to various ER markers in U2OS cells. Using immunofluorescence and confocal fluorescence microscopy, the localization of stably expressed REEP1-mEm or -mCh was compared with that of the endogenous ER membrane protein calnexin (αCNX), the endogenous tubular ER protein Reticulon-4 (αRtn4), an endogenous ER luminal marker (αKDEL), stably expressed ER membrane marker mScarlet-Sec61β (RFP-Sec61β), and/or a stably expressed ER luminal marker (ssRFP-KDEL). As controls, REEP5-mEm , REEP5-mCh, or RFP-Sec61β were also compared to the indicated ER markers. Localizations were analyzed in either fed or starved conditions (- or + 30 min EBSS). Means and standard deviations are indicated. Note that all mean Pearsons' values comparing REEP1 and all ER markers are below 0.5, indicating low colocalization. n, 74-152 cells/sample. P-values were calculated using one-way ANOVA analysis, multiple comparisons (Sidak's method). ****, $p<0.0001$; ns, not significant. Exact n-values and p-values are listed in the Source Data file.

d, U2OS cells stably expressing REEP1-mCh (green in the overlay) were analyzed by indirect immunofluorescence with anti-Rtn4 antibodies (αRtn4, magenta in the overlay), a tubular ER marker, and confocal fluorescence microscopy.

e, U2OS cells stably expressing REEP1-mEm and RFP-Sec61β were immunostained with αtubulin antibodies to visualize microtubules. Right panels, overlays of REEP1-mEm (green) and αtubulin (blue); bottom row, magnifications of the boxed regions. Scale bar, whole cell, 10 μm; magnification, 2 μm.

f, As in e, but after 30 min treatment with the microtubule polymerization inhibitor nocodazole. Right panel shows the overlay between REEP1-mEm (green) and RFP-Sec61β (magenta). Note the tubular ER has retracted from the periphery but the REEP1-mEm punctae remain.

g, Live U2OS cells stably expressing REEP1-mCh (magenta) were stained with DiOC6 (green), a lipophilic dye which stains bulk ER and mitochondrial membranes. The REEP1-mCh look 'fuzzier' than in fixed cells because they rapidly move during the exposure. Note the majority of REEP1-mCh punctae do not colocalize with ER membranes. Bottom row shows magnifications of the boxed region.

Arrowheads indicate REEP1-mCh punctae that align with (yellow) or are independent of (blue) the ER. n, nucleus; mito, mitochondria; PM, plasma membrane. Scale bars, whole cell, 10 μm; magnification, 2 μm.
h, As in g, but after 30 min treatment with nocodazole.

Supplementary Figure 3

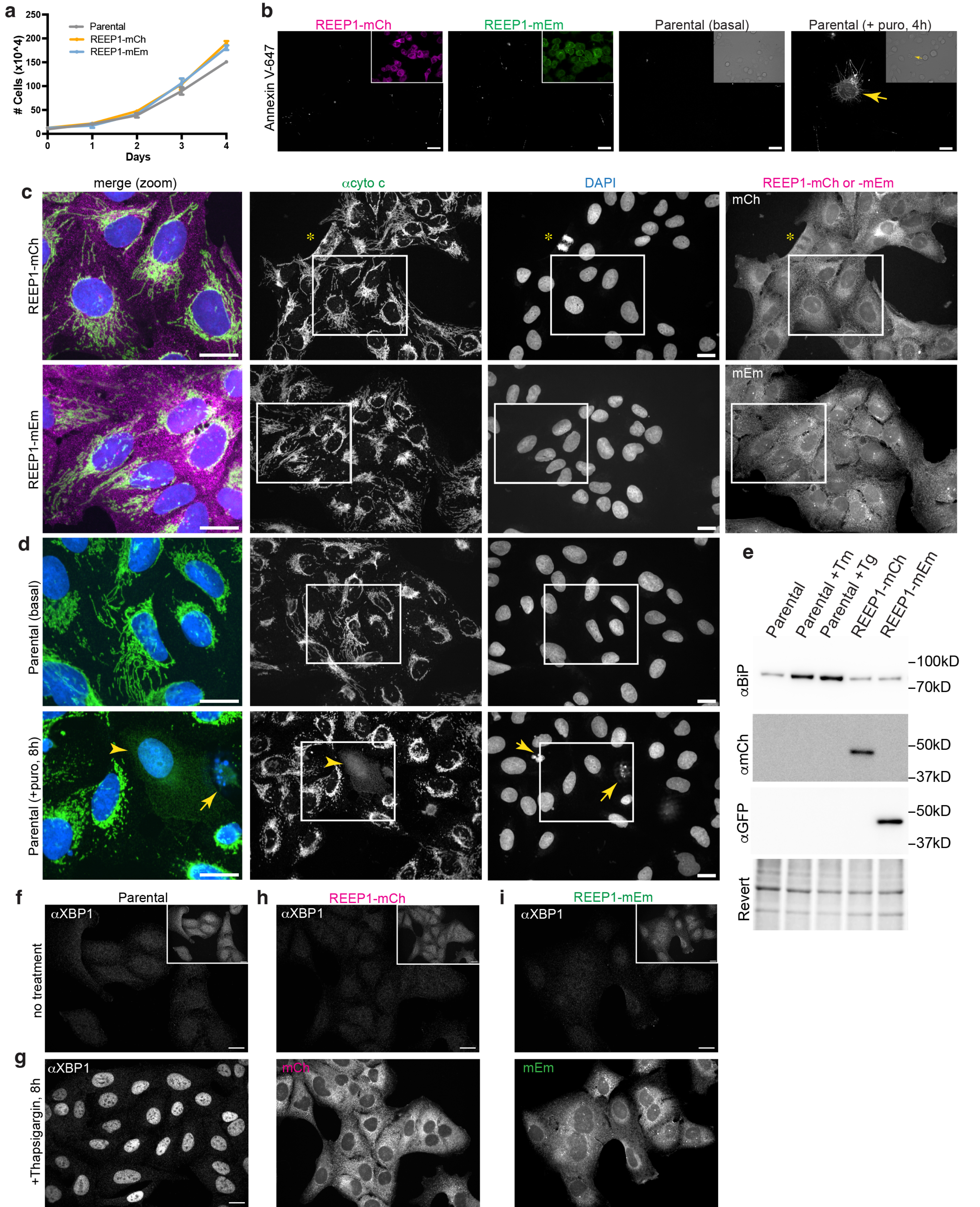

Supplementary Figure 3. U2OS cells stably expressing REEP1-mCh or -mEm grow normally and do not show elevated levels of apoptosis or ER stress.

a, Growth curves of parental U2OS cells and those stably expressing REEP1-mCh or REEP1-mEm. Cells were plated into three sets of 10 cm^2 dishes on day 0 and counted every 24 h. Shown are means and standard deviations. Exact values are listed in the Source Data file.

b, Live parental U2OS cells or those stably expressing REEP1-mCh (magenta) or REEP1-mEm (green) were grown under normal conditions, stained with the early apoptosis indicator Annexin V-647, and imaged with confocal microscopy. Parental cells were also treated with puromycin for 4 h to induce apoptosis. Arrow points to an apoptotic cell. Images were acquired with identical exposure settings and displayed with the same scaling. Insets show either fluorescence (mEm or mCh) or brightfield (parental) to indicate the presence of cells. Scale bars, 20 μm.

c, U2OS cells stably expressing REEP1-mCh (top row) or REEP1-mEm (bottom row) grown under normal conditions were stained with anti-cytochrome c (αcyto c) antibodies and the DNA dye DAPI to test for signs of apoptosis. Mitochondrial cytochrome c becomes cytosolic and nuclei bleb during late apoptosis. Left panel shows an enlarged, merged image (αcyto c, green; REEP1-mCh or mEm, magenta; DAPI, blue) of the boxed region. Note that all cells expressing REEP1-mCh and REEP1-mEm have mitochondria-localized cytochrome c and normal mitochondrial and nuclear morphologies. Asterisk indicates a mitotic cell in anaphase. Scale bars, 10 μm.

d, As in c, but with parental U2OS cells analyzed under basal conditions (top row) or treated with 1 μg puromycin for 8 h to induce apoptosis (bottom row). In puromycin-treated cells, arrowhead points to a cell with cytosolic cytochrome c and arrows point to cells with nuclear blebbing.

e, Lysates from U2OS cells stably expressing REEP1-mCh or REEP-mEm were analyzed for ER stress response induction by immunoblotting for BiP (αBiP). Lysates were compared to parental cells grown under basal conditions or treated for 8 h with the ER stressors tunicamycin (Tm) or thapsigargin (Tg). Revert700 (Revert) staining was used to demonstrate equal loading.

f, Parental U2OS cells grown under basal conditions were immunostained for XBP1 (αXBP1) and imaged with confocal microscopy. XBP1 is an ER stress transcription factor that localizes to the nucleus under stress. Scale bar, 10 μm.

g, As in f, but with cells treated for 8 h with thapsigargin. Note the prominent nuclear XBP1 localization.

h, As in f, but with U2OS cells stably expressing REEP1-mCh.

i, As in f, but with U2OS cells stably expressing REEP1-mEm.

All αXBP1 images in f-i were acquired and scaled identically for display. Insets in f, h-i are of the same αXBP1 images whose gain has been equivalently and linearly increased to indicate the presence of cells.

Supplementary Figure 4

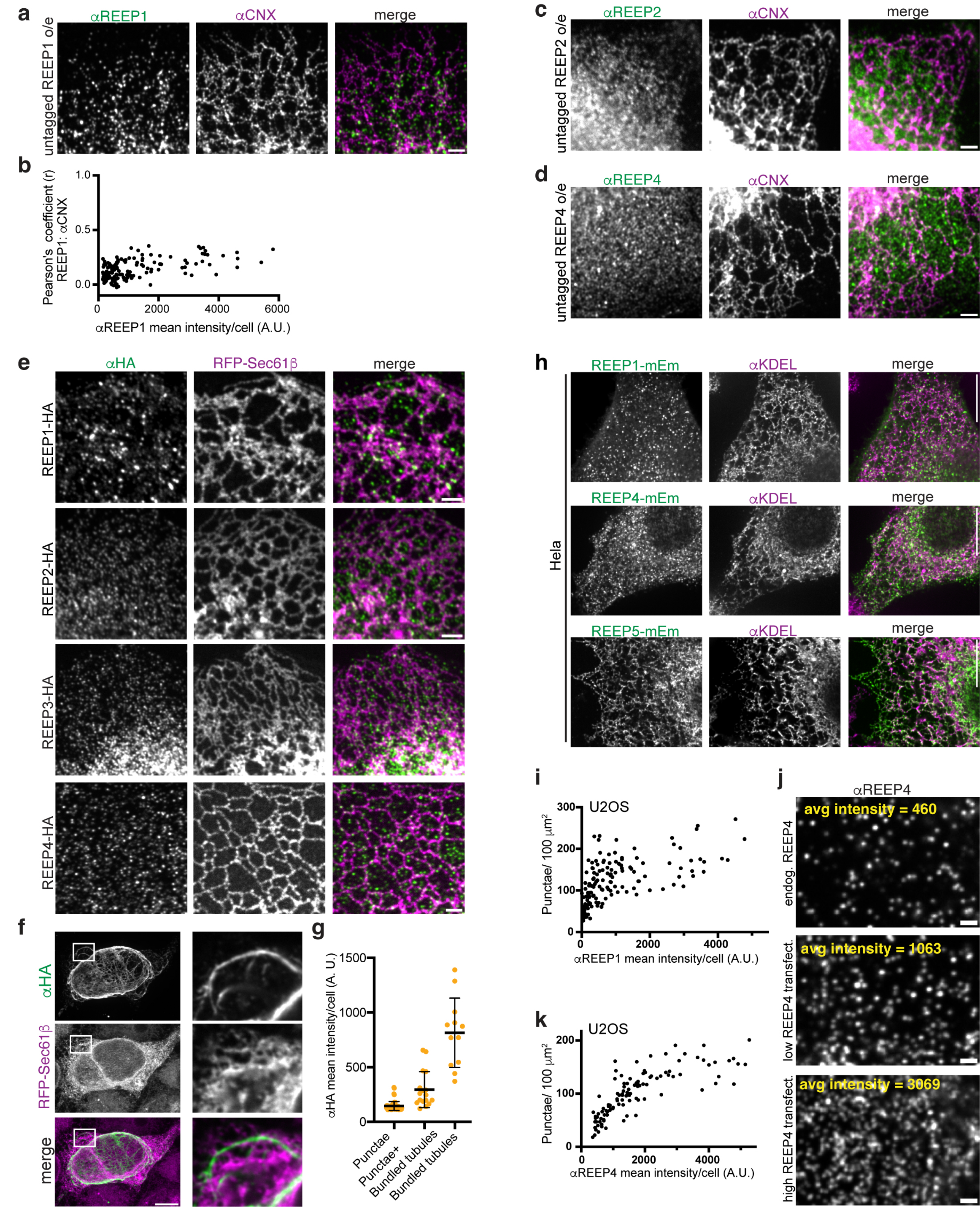

Supplementary Figure 4. REEP1-4 proteins localize to punctae independent of tags, expression levels, or cell line background.

a, U2OS cells were transfected with untagged REEP1 and immunostained with antibodies against REEP1 (αREEP1) and the ER membrane marker calnexin (αCNX). Scale bar, 2 μm.

b, Relative REEP1 localization compared to the bulk ER was analyzed across different REEP1 expression levels. Pearson's coefficients (r) comparing αREEP1 and αCNX localizations in REEP1-transfected cells were analyzed and plotted against the mean fluorescence intensities of each cell's αREEP1 signal. n, 151 cells. All values are listed in Source Data.

c, As in a, but with transfection of an untagged REEP2 construct and immunostaining with αREEP2 antibodies. Scale bar, 2 μm.

d, As in a, but with transfection of an untagged REEP4 construct and αREEP4 immunostaining.

e, U2OS cells stably expressing the ER marker RFP-Sec61β were transfected with HA-tagged REEP1, REEP2, REEP3, or REEP4 and analyzed by indirect immunofluorescence with anti-HA (αHA) antibodies and confocal fluorescence microscopy. Scale bars, 2 μm.

f, As in e, but with a cell expressing high levels of REEP1-HA that has a bundled tubular localization. Left column shows a maximal projection of a z-series through the entire cell volume. Right column shows a magnified, single focal plane view of the boxed region. Scale bar, 10 μm.

g, Quantification of REEP1-HA localization phenotype relative to expression levels of transiently transfected cells, as in e. The localization patterns were grouped into punctae only, punctae and bundled tubules, or predominantly bundled tubules, and compared to relative expression levels of REEP1-HA, as measured by mean αHA fluorescence intensity of the cell. Shown are means and standard deviations. Exact values are listed in Source Data.

h, Hela cells stably expressing REEP1-mEm, REEP4-mEm, or REEP5-mEm were immunostained with antibodies against the ER luminal marker αKDEL. Scale bars, 10 μm.

i, U2OS cells were transfected with untagged REEP1 as in d, immunostained with αREEP1, and the number of αREEP1 positive punctae was quantified in a region of the cell. The number of punctae/100 μm^2 was graphed against relative REEP1 expression levels, as measured by mean fluoresence intensities of the cell. Each data point represents measurements from one cell. n, 139 cells. All values are listed in Source Data.

j, U2OS cells were transiently transfected with REEP4-HA plasmid and immunostained with αREEP4 antibodies. Shown are regions from cells with different REEP4 expression levels (endogenous, low transfected, and high transfected). Inset values show the cell's mean αREEP4 fluorescence intensities.

k, As in i, but for REEP4-HA transfected U2OS cells shown in j. n, 104 cells.

Supplementary Figure 5

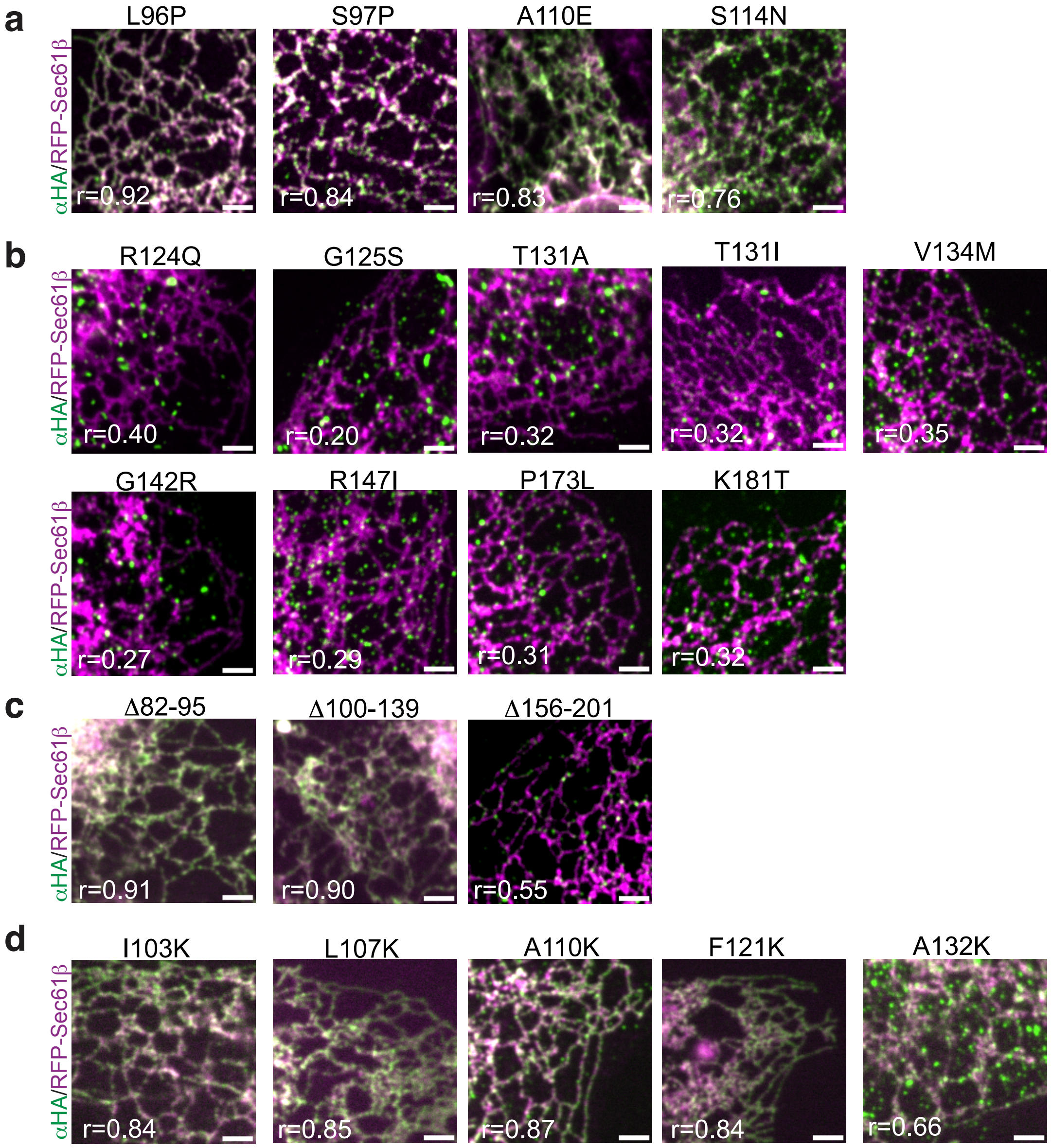

Supplementary Figure 5. Mutations in the C-terminal APH relocalize REEP1 to the bulk ER.

a, Constructs encoding HA-tagged REEP1 mutants containing putative HSP-linked point mutations L96P, S97P, A110E, or S114N were transfected into U2OS cells stably expressing RFP-Sec61β, and the mutants' localizations were analyzed by immunostaining with αHA antibodies. Shown are merged images between αHA (green) and RFP-Sec61β (magenta). r, Pearson's correlation coefficients (r) between αHA and RFP-Sec61β signals. Note that these mutations relocalize REEP1 to the bulk ER. Scale bar, 2 μm.

b, As in a, but with REEP1 constructs containing putative HSP-linked point mutations that do not affect REEP1 localization to vesicles.

c, As in a, but with REEP1 constructs with different C-terminal deletions (Δ82-95, Δ100-139, or Δ156-201).

d, As in a, but with REEP1 constructs containing single lysine mutations (I103K, L107K, A110K, F121K, and A132K) introduced into the APH-C.

Supplementary Figure 6

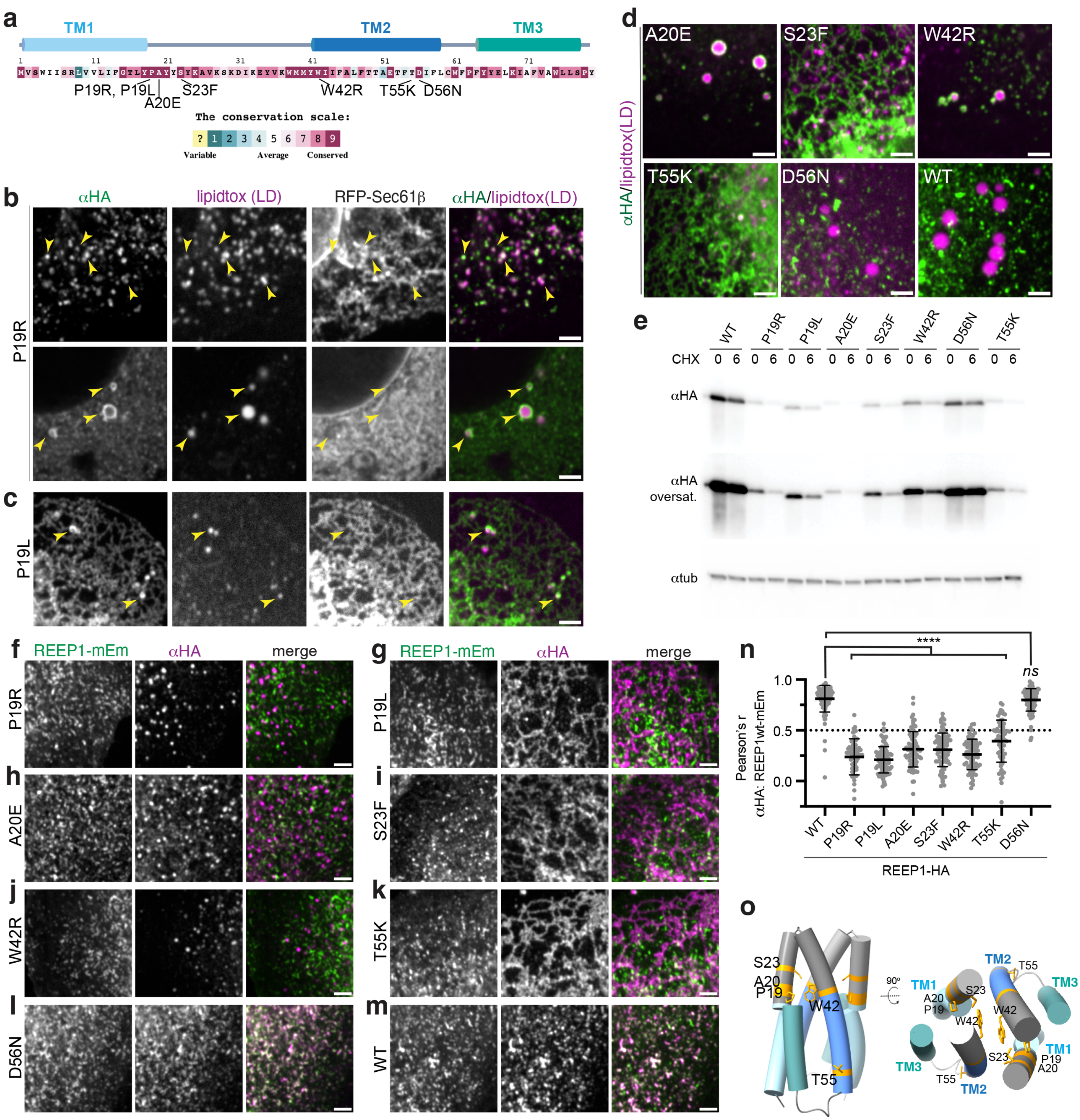

Supplementary Figure 6. The TM domain of REEP1 is important for vesicle localization.

a, Schematic of the TM domain of REEP1. Primary sequence conservation was analyzed using ConSurf software[1] (http://consurf.tau.ac.il), and disease mutations in HSP are indicated. The color scale indicates relative evolutionary conservation of each residue (teal, least conserved, to magenta, conserved). TM segments are indicated above the primary sequence.

b, U2OS cells stably expressing RFP-Sec61β were transfected with a HA-tagged REEP1 construct carrying the P19R disease mutation, and localization was determined by αHA immunostaining and confocal microscopy. Lipid droplets (LD) were visualized with Lipidtox. Right panels show overlays of αHA (green) and lipidtox (magenta). Arrowheads point to examples of colocalized αHA and LD signals. Top row shows a cell with numerous, small REEP1(P19R)-HA punctae; the bottom row shows a cell with REEP1(P19R)-HA at the rim of a larger LD. Scale bar, 2 µm.

c, As in b, but with a REEP1-HA construct carrying the P19L disease mutation.

d, REEP1-HA constructs carrying the A20E, S23F, W42R, T55K, D56N, or no (WT) disease mutation were transfected into U2OS cells and analyzed as in b. Shown are the overlays of αHA (green) and Lipidtox (magenta).

e, The stabilities of REEP1 TM domain mutants were analyzed by cycloheximide-chase experiments. Cells transfected with equivalent DNA amounts of wild-type or mutant REEP1-HA were collected at timepoint 0 or after 6h cycloheximide (CHX) treatment, and lysates were analyzed by immunoblotting with αHA and anti-α tubulin (loading control) antibodies. The middle blot shows the anti-HA blot linearly increased for gain (oversat.) to visualize the dimmer REEP1-HA mutant bands.

f-l, U2OS cells stably expressing REEP1-mEm were transfected with REEP1-HA carrying the indicated disease mutations, and localization was analyzed by anti-HA immunostaining and confocal fluorescence microscopy. Shown are overlays between REEP1-mEm (green) and αHA (magenta).

m, As in f-l, but with wild-type (WT) REEP1-HA.

n, Pearson's correlation coefficients were measured between the αHA and mEm signals of samples as analyzed in f-m. Shown are means and standard deviations. n, 65-100 cells/sample. P-values were calculated using one-way ANOVA analysis, multiple comparisons (Dunnett's method). ****, $p<0.0001$; ns, not significant. Exact n- and p-values are listed in Source Data.

o, Alphafold-predicted model of the REEP1 dimer interface. For clarity, residues above the dashed line were omitted in the topdown view. Residues whose mutation causes relocalization are shown in yellow.

Supplementary Figure 7

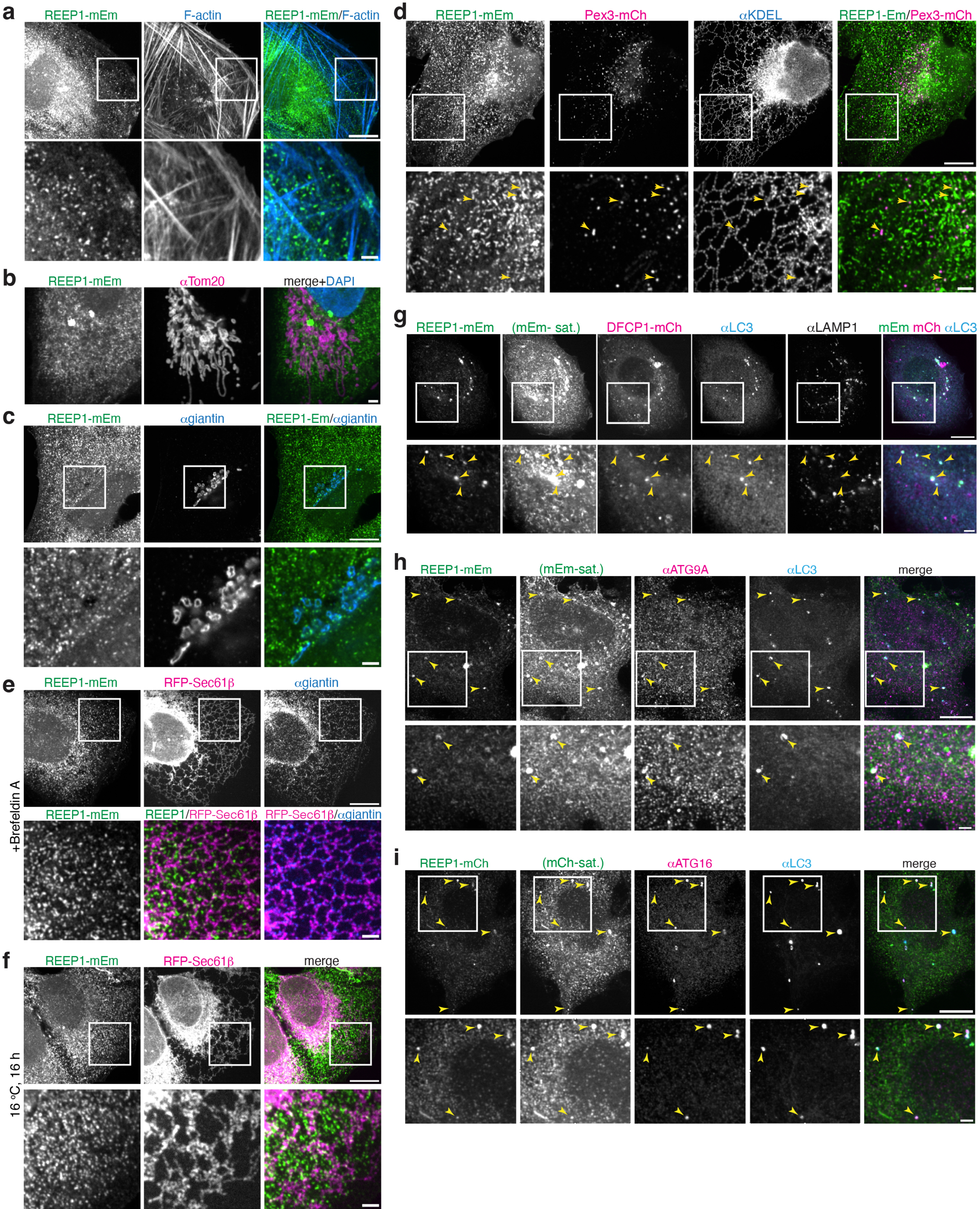

Supplementary Figure 7. REEP1 vesicles are distinct from other organelles and insensitive to inhibition of ER to Golgi transport.

a, U2OS cells stably expressing REEP1-mEm (green) were stained with phalloidin to visualize F-actin (blue) and imaged by confocal microscopy. Right panels show overlays. Bottom row shows magnifications of the boxed region. Scale bar for whole cell, 10 μm; magnification, 2 μm.

b, U2OS cells stably expressing REEP1-mEm was immunostained with Tom20 antibodies to visualize mitochondria. Right panel shows the overlay, with the addition of a DAPI image (blue). Scale bar, 2 μm.

c, As in b, but with cells immunostained with antibodies against giantin (blue) to visualize the Golgi. Bottom row shows magnifications of the boxed region. Scale bars for whole cell, 10 μm; magnification, 2 μm.

d, U2OS cells stably co-expressing REEP1-mEm and the peroxisomal protein Pex3-mCh were stained with αKDEL antibodies. Right panel shows the overlay between REEP1-mEm (green) and Pex3 (magenta). Bottom row shows enlargements of the boxed region. Arrowheads mark examples of Pex3-mChstructures that overlap with REEP1-mEm. Scale bars, whole cell, 10 μm; magnification, 2 μm.

e, As in c, but with cells also stably expressing RFP-Sec61β (magenta) and treated with Brefeldin A to inhibit trafficking between ER and Golgi. Note that this inhibitor disperses the Golgi.

f, U2OS cells stably expressing REEP1-mEm (green) and RFP-Sec61β (magenta) were grown at 16 ºC for 16 h to block vesicle trafficking through the secretory pathway and analyzed with confocal microscopy. Bottom row, magnifications of boxed region. Scale bars, whole cell, 10 μm; magnification, 2 μm.

g, U2OS cells stably expressing REEP1-mEm were transfected with mCherry-DFCP1 (mCh-DFCP1), starved in EBSS for 30 min, and immunostained with anti-LC3 (αLC3) and anti-LAMP1 (αLAMP1) antibodies. DFCP1 marks sites for autophagosome formation on the ER, LC3 marks phagophore membranes, and LAMP1 is a lysosome marker. The merged image shows the overlay of REEP1-mEm (green), mCh-DFCP1 (magenta), and αLC3 (blue). Bottom row shows magnifications of the boxed regions; arrowheads mark examples of LC3-positive autophagosomes. Second to leftmost column shows the REEP1-mEm image where the gain has been linearly increased (mEm-sat.) to visualize the dimmer REEP1-mEm punctae, which are all independent of autophagosomes. Scale bar, whole cell, 10 μm; magnification, 2 μm.

h, REEP1-mEm (green) expressing U2OS cells were starved in EBSS in the presence of MRT68921, an autophagy inhibitor that leads to enrichment of stalled early phagophore structures[2], and stained with antibodies against LC3 (αLC3, blue), the early autophagy marker ATG9A (αATG9A, magenta), and the lysosomal marker αLAMP1. Bottom row shows magnifications of the boxed region and arrowheads mark

examples of ATG9A-/LC3-positive phagophore structures. Scale bar, whole cell, 10 μm; magnification, 2 μm. Note that the majority of REEP1-mEm punctae do not colocalize with αLC3 or αATG9A.

i, As in h, but with REEP1-mCh (green) expressing cells stained with the antibodies against LC3 (blue) and the early autophagy marker ATG16 (αATG16, magenta).

Supplementary Figure 8

a

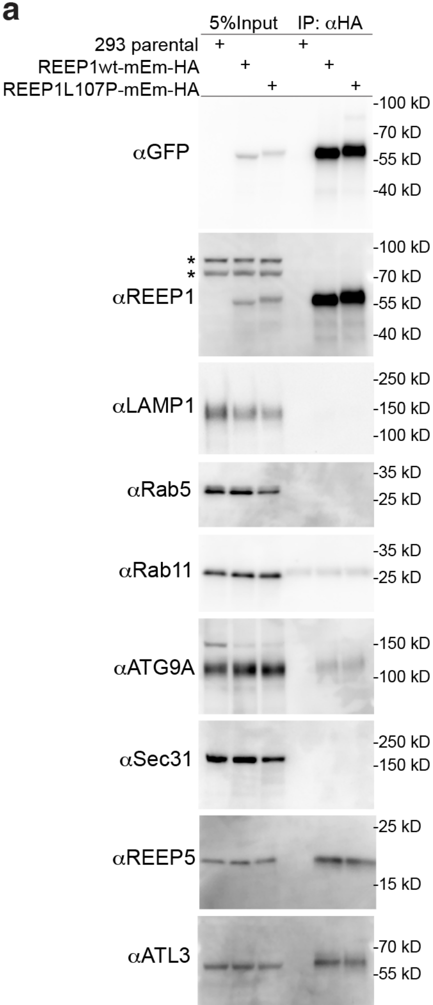

b

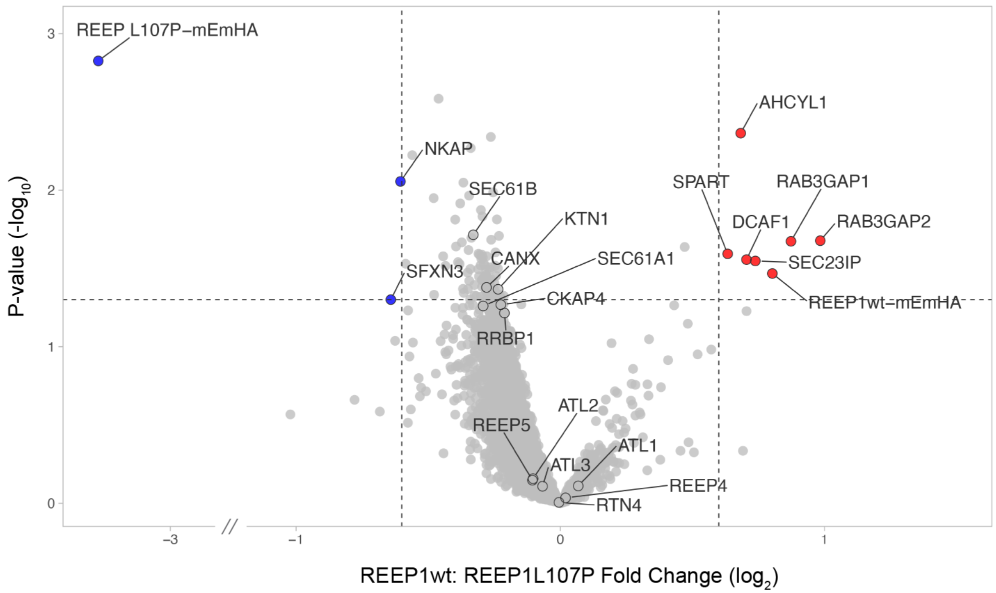

Supplementary Figure 8. Comparison of membrane compositions containing REEP1wt- or REEP1L107P.

a, Cell lysates were prepared from parental 293 Flp-In cells or cells expressing REEP1wt-mEm-3xHA or REEP1L107P mEm-3xHA. The lysates were either analyzed directly by immunoblotting (Input) or were subjected to immunoprecipitation with magnetic beads containing anti-HA antibodies in the absence of detergent. Input and eluates from the beads were analyzed by immunoblotting with various antibodies: anti-GFP, anti-REEP1, anti-LAMP1 (lysosomes), anti-Rab5 (early endosomes), anti-Rab11 (recycling endosomes), anti-ATG9A (early autophagosomal marker), anti-Sec31 (COPII), anti-REEP5 (tubular ER), or anti-ATL3 (tubular ER). *, nonspecific bands.

b, The relative enrichment of proteins from membrane immunoprecipitation experiments in a were compared by quantitative proteomics using TMT-labeling. Each datapoint in the volcano plot represents a protein for which the ratio of its abundance in the REEP1 wt vs. REEP1 L107P samples is given (Fold Change, $\log_2$), as well as p-values ($-\log_{10}$) derived from multiple two-tailed unpaired t tests with Welch's correction. Values were calculated from six biological replicates, and cut-offs are set for p-values ($-\log_{10}$) at 1.3 and fold change at $\log_2 = \pm 0.6$. Some representative proteins in the ER are also indicated. The proteins shown in red are enriched in REEP1 wt membranes; in blue, enriched in REEP1 L107P membranes. Exact p-values are provided in Supplementary Data 1.

Supplementary Figure 9

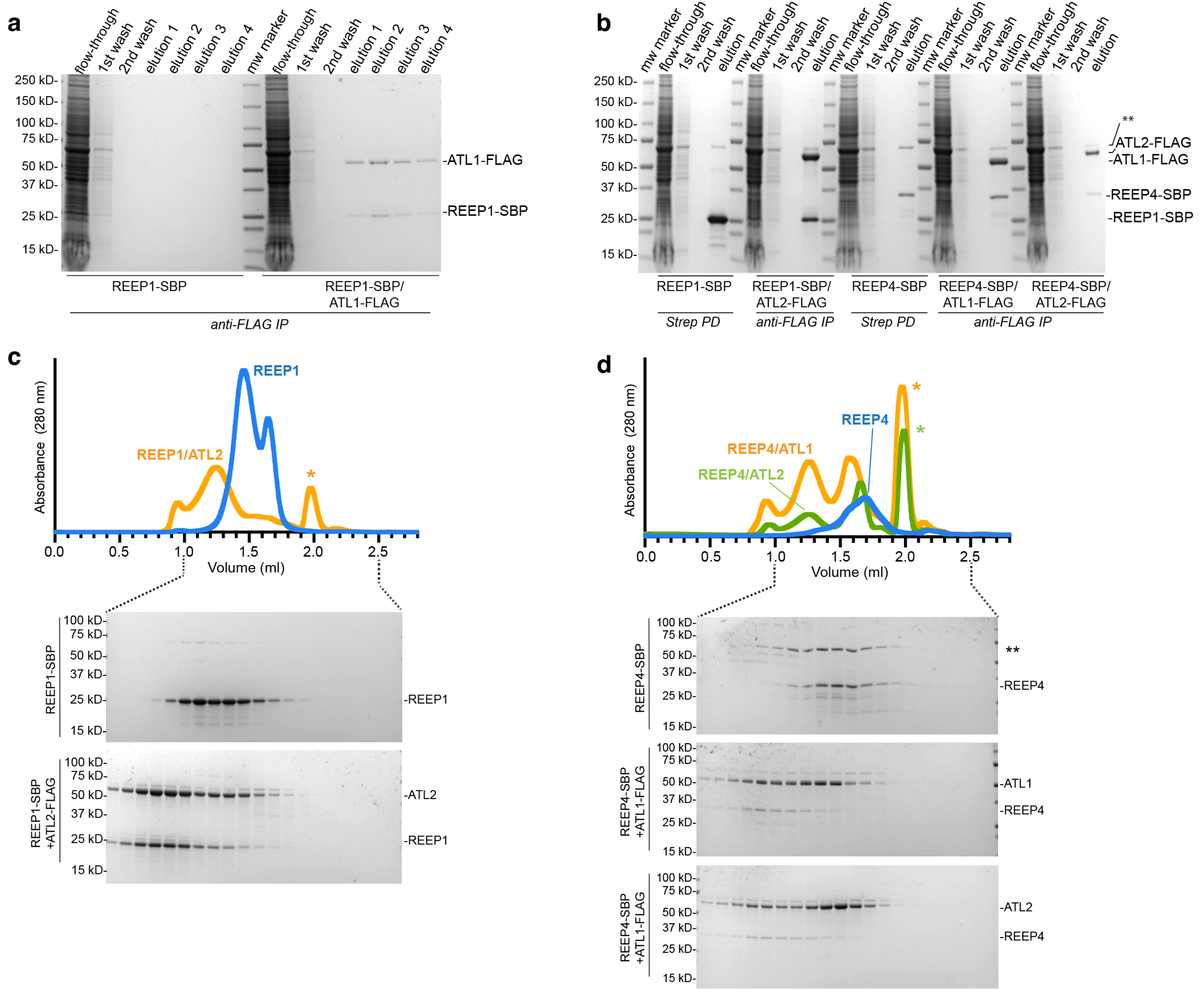

Supplementary Figure 9. REEP1 and REEP4 interaction with ATL proteins.

a, Expi293 cells were transfected with REEP1-SBP alone or with ATL1-FLAG, solubilized in digitonin, and subjected to immunoprecipitation (IP) with anti-FLAG conjugated beads. The beads were washed twice (wash 1, 2) and bound material was eluted with FLAG peptide in four fractions. All samples were analyzed by SDS-PAGE and Coomassie blue staining. Note that REEP1-SBP co-elutes with ATL1-FLAG.

b, As in a, but with lysates co-transfected with REEP1-SBP and ATL2-FLAG, REEP4-SBP and ATL1-FLAG, or REEP4-SBP and ATL2-FLAG. Lysates transfected with REEP1-SBP or REEP4-SBP alone were similarly subjected to pulldowns (PD) with streptavidin-conjugated beads and eluted with biotin. The band at ~70 kD (**) is the cytosolic molecular chaperone Hsp70, as confirmed by tandem mass spectrometry.

c-d, The eluted material from b was analyzed by size-exclusion chromatography. Eluted fractions were analyzed by SDS-PAGE and Coomassie blue staining. The elution peaks marked with (*) correspond to the 3xFLAG peptide used for ATL1/2-FLAG IPs; the 70 kD band (**) is the contaminant Hsp70. Note that these samples were analyzed on a different chromatography system than in Fig 6a, which results in different elution volumes.

Supplementary Figure 10

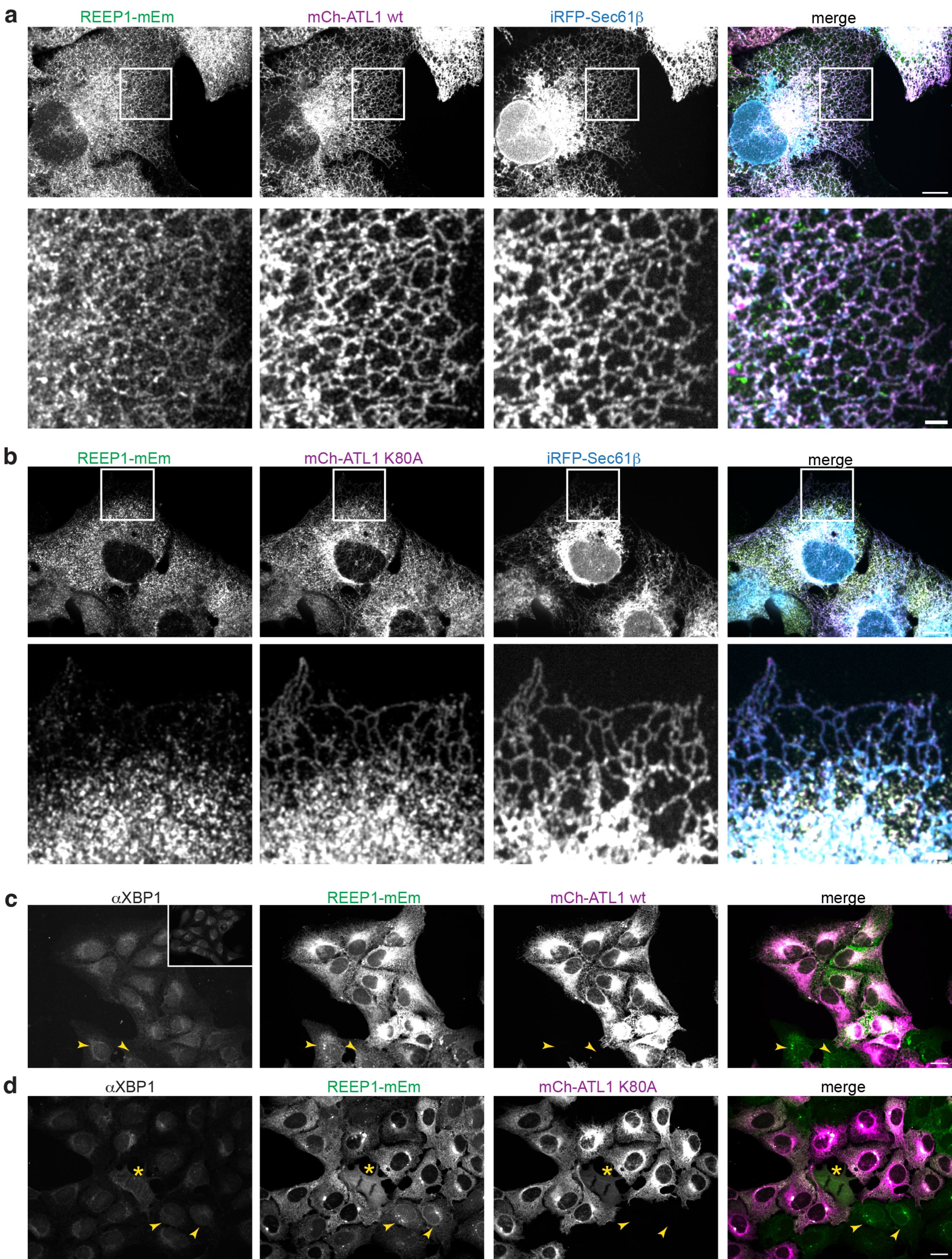

Supplementary Figure 10. U2OS cells stably expressing wild-type or mutant ATL1 have intact ER and do not show signs of ER stress.

a, U2OS cells stably expressing REEP1-mEm (green), mCh-ATL1 wt (magenta), and iRFP-Sec61β (blue) were fixed and imaged by confocal microscopy. Bottom row, magnifications of the boxed region. Scale bars, whole cell, 10 μm; magnification, 2 μm.

b, As in a, but with cells stably co-expressing mCh-ATL1 K80A instead of ATL1 wt. Note that both REEP1-mEm and mCh-ATL1 K80A are enriched in ER-independent punctae, while the ER marked by iRFP-Sec61β remains intact.

c, U2OS cells stably co-expressing REEP1-mEm (green) and mCh-ATL1 wt (magenta) were immunostained with αXBP1 antibodies to test for ER stress response induction. Arrows mark cells that are not expressing mCh-ATL1. Inset is of U2OS parental cells grown at basal conditions and immunostained with αXBP1 antibodies. Scale bar, 20 μm.

d, As in c, but with cells co-expressing mCh-ATL1 K80A instead of ATL1 wt. Asterisk marks a mitotic cell.

αXBP1 images in c-d were all imaged and scaled identically for display. Note that there is no difference in αXBP1 intensity or localization across all cells in c-d.

Supplementary Figure 11

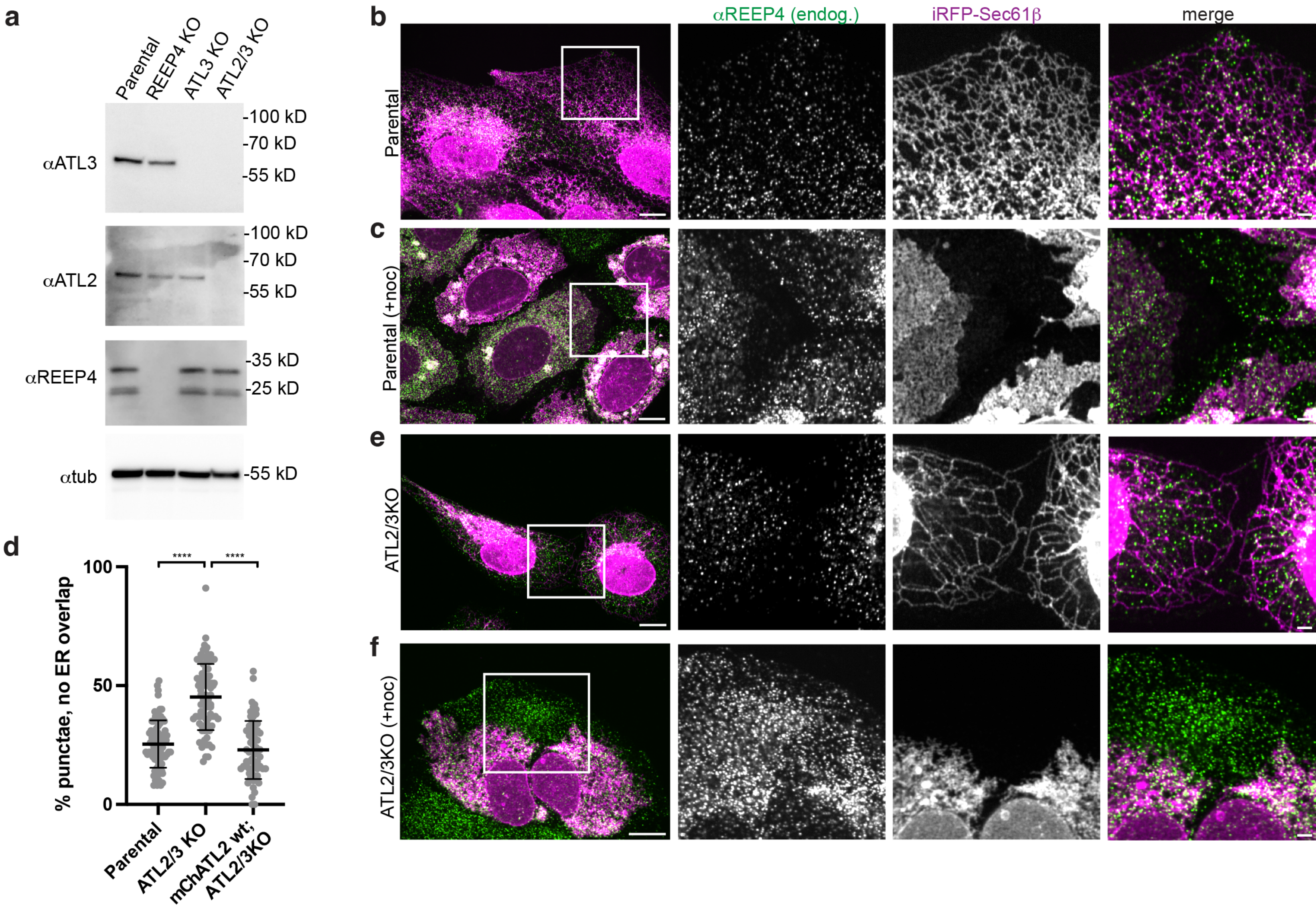

Supplementary Figure 11. Endogenous REEP4 punctae localization is dependent on ATLs.

a, Immunoblots of cell lysates from parental U2OS cells or from those lacking REEP4 (REEP4 KO), ATL3 (ATL3 KO), or ATL2 and ATL3 (ATL2/3 KO), probed with antibodies against ATL2, ATL3, and REEP4. Blotting with αtubulin serves as a loading control.

b, Parental U2OS cells stably expressing the ER marker iRFP-Sec61β (magenta) were immunostained with αREEP4 antibodies (green) and imaged with confocal microscopy. Right panels show enlargements of the boxed region. Scale bars, whole cell, 10 μm; enlargement, 2 μm.

c, As in b, but with ATL2/3 KO cells.

d, Quantification of endogenous REEP4 punctae that do not overlap with the ER, as imaged in b-c. The total number of REEP4 punctae were counted in cell regions where the ER displayed a clear reticulated network, and the percentage of punctae that does not overlap with iRFP-Sec61β signal was quantified per cell. Parental U2OS cells, ATL2/3 double KO cells, and ATL2/3 KO cells stably expressing mCh-ATL2 wt were analyzed. n, 84, parental; 86, ATL2/3 KO; 63, mChATL2 wt, ATL2/3 KO. Shown are means and standard deviations. P-values were calculated using one-way ANOVA, multiple comparisons (Dunnett's method). ****, $p<0.0001$. Exact p-values are listed in Source Data. The parental sample is the same sample graphed in Supplementary Fig. 1c.

e, As in b, but of cells treated for 30 min with nocodazole.

f, As in c, but of cells treated for 30 min with nocodazole.

Disease mutation	Mutation location within protein	Provenance	Disease, Clinical Significance
REEP1(Δ113-201)*	APH-C + disordered C-term tail	Hewamadduma, et al, 2009.	HSP, Pathogenic
REEP1(Δ102-139)	APH-C	Beetz et al, 2012.	HMN5b, Pathogenic
REEP1(P19L)	TM domain	Goizet et al, 2011.	HSP, Pathogenic
REEP1(P19R)	TM domain	Beetz et al, 2008.	HSP, Pathogenic
REEP1(A20E)	TM domain	Zuchner et al, 2006.	HSP, Pathogenic
REEP1(S23F)	TM domain	Goizet et al, 2011.	HSP, Pathogenic
REEP1(W42R)	TM domain	Goizet et al, 2011.	HSP, Pathogenic
REEP1(T55K)	TM domain	Schlang, et al, 2008.	HSP, Pathogenic/uncertain significance
REEP1(D56N)	TM domain	Goizet et al, 2011.	HSP, Uncertain significance
REEP1(L96P)	APH-C	ClinVar accession # VCV000534215.6	HSP, Uncertain significance
REEP1(S97P)	APH-C	ClinVar accession # VCV000578828.5	HSP, Uncertain significance
REEP1(L107P)	APH-C	Schlang et al, 2008.	HSP, Pathogenic
REEP1(A110E)	APH-C	ClinVar accesssion # RCV001253597.1	HSP, Uncertain significance
REEP1(S114N)	APH-C	ClinVar accession # RCV001043828.1	HSP, Uncertain significance
REEP1(L118R)	APH-C	ClinVar accession # RCV000641691.5	HSP, Uncertain significance
REEP1(R124Q)	APH-C	ClinVar accession # RCV000701587.3	HSP, Uncertain significance
REEP1(G125S)	APH-C	ClinVar accession # VCV000566687.5	HSP, Uncertain significance
REEP1(N127D)	APH-C	ClinVar accession # VCV000896929.4	HSP, Uncertain significance
REEP1(T131A)	APH-C	ClinVar accession # RCV000698241.5	HSP, Uncertain significance
REEP1(T131I)	APH-C	ClinVar accession # RCV000641682.5	HSP, Uncertain significance
REEP1(V134M)	APH-C	ClinVar accession # RCV000641681.5	HSP, Uncertain significance
REEP1(G142R)	between APH-C and disordered C-term tail	ClinVar accession # RCV000204692.4	HSP, Uncertain significance
REEP1(R147I)	between APH-C and disordered C-term tail	ClinVar accession # RCV000473114.7	HSP, Uncertain significance
REEP1(P173L)	between APH-C and disordered C-term tail	ClinVar accession # RCV000204692.4	HSP, Uncertain significance/likely benign
REEP1(K181T)	between APH-C and disordered C-term tail	ClinVar accession # RCV000641690.5	HSP, Uncertain significance
REEP2(Δ111-252)**	APH-C + disordered C-term tail	ClinVar accession # VCV001029275.1	HSP, Likely pathogenic

*Truncated protein is caused by a nonsense mutation leading to a premature stop codon (R113X)
**Truncated protein is caused by a nonsense mutation leading to a premature stop codon (R111X)

Supplementary Table 1. REEP1 and REEP2 disease-associated mutations used in this study.
Listed are the mutations' domain location within the protein, provenance, and established clinical significance.

Supplementary References

1 Ashkenazy, H. *et al.* ConSurf 2016: an improved methodology to estimate and visualize evolutionary conservation in macromolecules. *Nucleic Acids Res* **44**, W344-350 (2016). https://doi.org:10.1093/nar/gkw408

2 Zachari, M., Longo, M. & Ganley, I. G. Aberrant autophagosome formation occurs upon small molecule inhibition of ULK1 kinase activity. *Life Sci Alliance* **3** (2020). https://doi.org:10.26508/lsa.202000815
