# Supplementary material for: The membrane curvature-inducing REEP1-4 proteins generate an ER-derived vesicular compartment
Source: Nat Commun. 2024 Oct 5;15:8655. doi: 10.1038/s41467-024-52901-6 (PMC11455953; doi:10.1038/s41467-024-52901-6)
Supplement: Supplementary file 2 — Description of Additional Supplementary Information [file 41467_2024_52901_MOESM2_ESM.docx]

**Description of Additional Supplementary Files**

File Name: Supplementary Data 1

Description: Quantitative proteomics comparing REEP1 wt- and REEP1 L107P- membranes. Mass spectrometry analysis of TMT-labeled immunoprecipitated membranes isolated from 293 Flp-In cells expressing REEP1wt-mEmHA or REEP1L107P-mEmHA. Listed are all identified proteins, their peptide count, scaled signal-to-noise ratios, means, fold changes (FC), and p-values calculated from multiple, twotailed unpaired t-tests (Welch’s correction) from six biological replicates. Values are plotted in Fig S8b

File Name: Supplementary Data 2

Description: DNA plasmids, antibodies, reagents, and cell lines used in this study.

File Name: Supplementary Video 1

Description: REEP1mEm punctae are dynamic in live cells. Live U2OS cells stably expressing REEP1-mEm (green) and mScarlet-Sec61β (magenta) were imaged for 2 min at 2 sec intervals. Video speed has been increased 20x. Scale bar, 2 μm. Note that the majority of REEP1 punctae move independently of the ER, but some remain associated with ER tubules. Arrowheads indicate examples of ER-associated REEP1-mEm punctae.

File Name: Supplementary Video 2

Description: REEP1-mCh punctae are dynamic and associate with ER in live cells. Live U2OS cells stably expressing REEP1-mCh (magenta) and stained with DiOC6 (green) were imaged as in Supplementary Video 1. Most REEP1-mCh punctae move rapidly and independently of the ER, but some are associated with ER tubules. Examples of ERassociated punctae are highlighted. Note one punctum travels with a growing ER tubule tip. Scale bar, 2 μm.

File Name: Supplementary Video 3

Description: REEP1 punctae associate with ER tubule tips. Live U2OS cells stably expressing REEP1-mCh were stained with DiOC6 and imaged as in Supplementary Video 2. Highlighted is an example of a REEP1-mCh punctum that localizes to and travels with a growing ER tubule tip. Scale bar, 2 μm.

File Name: Supplementary Video 4

Description: REEP1-mCh punctae dissociation from the ER. Live U2OS cells stably expressing REEP1-mCh were stained with DiOC6 and imaged as in Supplementary Video 2. Highlighted is an example of a REEP1-mCh punctum that initially is associated with the ER but quickly detaches. Scale bar, 2 μm.

File Name: Supplementary Video 5

Description: mChATL1 K80A vesicles are dynamic and independent of bulk ER membranes. Live U2OS cells stably expressing REEP1-mEm, mCh-ATL1 K80A, and the ER marker iRFPSec61β were imaged with nodelay capture. Top row: Left panel shows REEP1-mEm (green), middle panel shows mCh-ATL1 K80A (magenta), and right panel shows iRFPSec61β (blue); bottom row, left panel shows merged timelapse of REEP1-mEm/iRFP-Sec61β and right panel shows merged timelapse of mCh-ATL1K80A. Note that ATL1 K80A punctate move dynamically and often independently from the bulk ER and that the bulk ER is intact. Scale bar, 10 μm.
