# Supplementary material for: The membrane curvature-inducing REEP1-4 proteins generate an ER-derived vesicular compartment
Source: Nat Commun. 2024 Oct 5;15:8655. doi: 10.1038/s41467-024-52901-6 (PMC11455953; doi:10.1038/s41467-024-52901-6)
Supplement: Supplementary file 10 — Reporting Summary [file 41467_2024_52901_MOESM10_ESM.pdf]

nature portfolio

Corresponding author(s): Yoko Shibata, Tom Rapoport

Last updated by author(s): Sep 20, 2024

Reporting Summary

Nature Portfolio wishes to improve the reproducibility of the work that we publish. This form provides structure for consistency and transparency in reporting. For further information on Nature Portfolio policies, see our Editorial Policies and the Editorial Policy Checklist.

Statistics

For all statistical analyses, confirm that the following items are present in the figure legend, table legend, main text, or Methods section.

n/a	Confirmed	
☐	☒	The exact sample size (*n*) for each experimental group/condition, given as a discrete number and unit of measurement
☐	☒	A statement on whether measurements were taken from distinct samples or whether the same sample was measured repeatedly
☐	☒	The statistical test(s) used AND whether they are one- or two-sided<br>*Only common tests should be described solely by name; describe more complex techniques in the Methods section.*
☒	☐	A description of all covariates tested
☐	☒	A description of any assumptions or corrections, such as tests of normality and adjustment for multiple comparisons
☐	☒	A full description of the statistical parameters including central tendency (e.g. means) or other basic estimates (e.g. regression coefficient) AND variation (e.g. standard deviation) or associated estimates of uncertainty (e.g. confidence intervals)
☐	☒	For null hypothesis testing, the test statistic (e.g. *F*, *t*, *r*) with confidence intervals, effect sizes, degrees of freedom and *P* value noted<br>*Give P values as exact values whenever suitable.*
☒	☐	For Bayesian analysis, information on the choice of priors and Markov chain Monte Carlo settings
☒	☐	For hierarchical and complex designs, identification of the appropriate level for tests and full reporting of outcomes
☐	☒	Estimates of effect sizes (e.g. Cohen's *d*, Pearson's *r*), indicating how they were calculated

Our web collection on statistics for biologists contains articles on many of the points above.

Software and code

Policy information about availability of computer code

Data collection	Metamorph v7.8, Nikon NIS-Elements v5.21.03, ImageQuant TL 10.1, Licor Acquisition, AMT imaging, Unicorn System Control v7
Data analysis	FIJI/ImageJ (1.54i), Microsoft Excel, Graphpad Prism (10.2.3), MSconvert

For manuscripts utilizing custom algorithms or software that are central to the research but not yet described in published literature, software must be made available to editors and reviewers. We strongly encourage code deposition in a community repository (e.g. GitHub). See the Nature Portfolio guidelines for submitting code & software for further information.

Data

Policy information about availability of data

All manuscripts must include a data availability statement. This statement should provide the following information, where applicable:

- Accession codes, unique identifiers, or web links for publicly available datasets
- A description of any restrictions on data availability
- For clinical datasets or third party data, please ensure that the statement adheres to our policy

Mass spectrometry data have been deposited to the ProteomeXchange Consortium via the PRIDE partner repository with the dataset identifier PXD055950 [https://www.ebi.ac.uk/pride/archive/projects/PXD055950]. Raw, whole cell fluorescence images used for display are deposited in figshare, and all other raw data are available upon request. Source data are provided with this paper.

Research involving human participants, their data, or biological material

Policy information about studies with human participants or human data. See also policy information about sex, gender (identity/presentation), and sexual orientation and race, ethnicity and racism.

Reporting on sex and gender	n/a
Reporting on race, ethnicity, or other socially relevant groupings	n/a
Population characteristics	n/a
Recruitment	n/a
Ethics oversight	n/a

Note that full information on the approval of the study protocol must also be provided in the manuscript.

Field-specific reporting

Please select the one below that is the best fit for your research. If you are not sure, read the appropriate sections before making your selection.

- [x] Life sciences
- [ ] Behavioural & social sciences
- [ ] Ecological, evolutionary & environmental sciences

For a reference copy of the document with all sections, see nature.com/documents/nr-reporting-summary-flat.pdf

Life sciences study design

All studies must disclose on these points even when the disclosure is negative.

Sample size	Power analysis was not performed to predetermine sample size. Sample sizes are consistent with field standards in cell biology.
Data exclusions	No data were excluded for analysis.
Replication	Each experiment was biologically replicated successfully twice or more, except for Fig S1b, bottom row; S3b, S5b, S6e, S7a, Fig S9b-d, and S10c-d, all of which were performed once.
Randomization	All starting groups were randomly assigned. Each group represents a distinct treatment or condition.
Blinding	Investigators were not blinded to group allocation during data collection or analysis. Phenotypic differences were clear amongst different samples, and double-blinding would be ineffective.

Reporting for specific materials, systems and methods

We require information from authors about some types of materials, experimental systems and methods used in many studies. Here, indicate whether each material, system or method listed is relevant to your study. If you are not sure if a list item applies to your research, read the appropriate section before selecting a response.

Materials & experimental systems

n/a	Involved in the study	
[ ]	[x]	Antibodies
[ ]	[x]	Eukaryotic cell lines
[x]	[ ]	Palaeontology and archaeology
[x]	[ ]	Animals and other organisms
[x]	[ ]	Clinical data
[x]	[ ]	Dual use research of concern
[x]	[ ]	Plants

Methods

n/a	Involved in the study	
[x]	[ ]	ChIP-seq
[x]	[ ]	Flow cytometry
[x]	[ ]	MRI-based neuroimaging

Antibodies

Antibodies used	anti-GFP (mouse monoclonal JL-8), Clontech #632381, lot A80341331:5000 dilution for WB; anti-mCherry (mouse monoclonal 1C51), Abcam #ab125096, lot GR3394424-1, 1:2500 dilution for WB; anti-REEP5 (rabbit monoclonal EP11115), Abcam #ab167405, lot GR163095-1, 1:400 dilution for IF, 1:5000 dilution for WB; anti-Calnexin (rabbit polyclonal), Abcam #ab22595, lot GR3424430-1 and lot 1013331-2, 1:400 dilution for IF; anti-Calnexin (mouse monoclonal), MBL #M178-3, lot unknown, 1:2000 dilution for WB; anti-REEP1 (rabbit polyclonal), ThermoFisher #PA5-63515, lot XC3518329, 1:200 dilution for IF; anti-REEP1 (rabbit polyclonal), Proteintech

#17988-1-AP, lot 00017226, 1:1000 dilution for WB; anti-HA (rat monoclonal 3F10), Roche/Millipore Sigma #11867423001, lot 66109200, 1:1000 dilution for IF; anti-REEP2 (mouse monoclonal S3260-2), ThermoFisher #MA5-27625, lot WE3281371, 1:200 dilution for IF; anti-REEP4 (rabbit polyclonal), Atlas/Millipore Sigma #HPA042683, lot R39936, 1:200 dilutionfor IF; 1:1000 dilution for WB; anti-tubulin (mouse monoclonal DM1A), Millipore Sigma #T6199, lot 116M4802V , 1:400 dilution for IF; 1:2000 dilution for WB; anti-KDEL (mouse monoclonal 1D5), MBL #M181-3, lot 004, 1:400 dilution for IF; anti-Rtn4 (rabbit polyclonal), Abcam #ab47085, lot GR3328033-2, 1:400 dilution for IF; anti-Sec31 (mouse monoclonal clone 32), Becton Dickinson #612351, lot 9311782, 1:400 dilution for IF, 1:1000 dilution for WB; anti-cytochrome c (mouse monoclonal 6H2;B4), Becton Dickinson #556432, lot 1295628, 1:1000 dilution for IF; anti-Tom20 (mouse monoclonal MABC1108), Becton Dickinson #612278, lot 120258, 1:500 dilution for IF; anti-LAMP1 (mouse monoclonal H4A3), Millipore Sigma #MABC1108, lot 4034342, , 1:1000 dilution for IF; 1:1000 dilution for WB; anti-giantin (GOLGB1; rabbit polyclonal), ThermoFisher #PA5-52841, lot WI3378416 , 1:400 dilution for IF; anti-ATG9A (rabbit monoclonal EPR2450(2)), Abcam # ab108338, lot GR3300372-4, 1:200 dilution for IF, 1:1000 dilution for WB; anti-ATG16L (rabbit polyclonal), MBL # PM040MS, lot 003, 1:200 dilution for IF; anti-LC3 (rabbit polyclonal), MBL #PM036, lot 035, 1:200 dilution for IF; anti-LC3 (mouse monoclonal clone 4E12), MBL # M152-3, lot 057, 1:200 dilution for IF with permeabilization of cells by digitonin; anti-PMP70 (mouse monoclonal clone 70-18), Millipore Sigma #SAB4200181, lot 0000157142, 1:400 dilution for IF; anti-Rab5 (rabbit monoclonal clone EPR5438), Abcam # ab109534, lot GR3264745-1, 1:1000 dilution for WB; anti-Rab11 (mouse monoclonal clone 47), Becton Dickinson #610656, lot 0037124, 1:1000 dilution for WB; anti-XBP1 (mouse monoclonal E8C2Z), Cell Signaling Technology #27901, lot 1, 1:200 dilution for IF; anti-ATL2 (rabbit polyclonal), Bethyl Laboratories # A303-333A-T lot 1, 1:1000 dilution for WB; anti-ATL3 (rabbit polyclonal), Proteintech #16921-1-AP, lot 00111325, 1:1000 dilution for WB (Suppl Figure 8), anti-ATL3 (rabbit polyclonal), ThermoFisher #PA5-98495, lot YD3884264A, 1:1000 dilution for WB (Suppl Figure 11); anti-BiP (GRP78; mouse monoclonal 1H11-1H7), ThermoFisher # MA5-27686, lot ZC4246731, 1:2000 dilution for WB; anti-HA (rat monoclonal 3F10), HRP-conjugated, Millipore Sigma #12013819001, lot 54193500, 1:5000 dilution for WB.

Validation

All primary antibodies were commercially obtained and were validated for specificity for WB/IF by the manufacturer. Data are provided on their websites. We provide additional specificity data for the primary antibodies in this manuscript: anti-GFP (mouse monoclonal, WB, Fig 1f, Fig S8a): anti-mCherry (mouse monoclonal, WB, Fig S3e); anti-REEP5 (WB, Fig 1f, Fig S8a; IF, Fig S1b); anti-Calnexin (rabbit polyclonal, IF, Fig 4g, 4h); anti-Calnexin (mouse monoclonal, WB, Fig 1f); anti-REEP1 (rabbit polyclonal, ThermoFisher, IF, Fig S1a,b (RNAi), Fig S4a,b (overexpression)), anti-REEP1 (rabbit polyclonal, Proteintech, WB, Fig S8a), anti-HA (rat monoclonal, IF, Fig 2); anti-REEP2 (mouse monoclonal, IF, Fig S1d, Fig S4c (overexpression)); anti-REEP4 (rabbit polyclonal, WB, Fig S11a, CRISPR-KO; IF, S1e, CRISPR-KO); anti-tubulin (WB, Fig 1F; IF, S2e-f); anti-KDEL (mouse monoclonal, IF, Fig 4, 6, S2a); anti-Rtn4 (rabbit polyclonal, Fig 2d); anti-Sec31 (mouse monoclonal, WB, Fig S8a; IF, Fig 5i); anti-cytochrome c (mouse monoclonal, IF, Fig S3c,d); anti-Tom20 (mouse monoclonal, IF, S7c); anti-LAMP1 (WB, Fig S8a; IF, Fig S7g); anti-giantin (GOLGB1; rabbit polyclonal, Fig S7c); anti-ATG9A (rabbit monoclonal, WB, Fig S8a; IF, Fig S7d); anti-ATG16L (rabbit polyclonal, IF, Fig S7i); anti-LC3 (rabbit polyclonal, IF, Fig S7g); anti-LC3 (mouse monoclonal, IF, Fig S7h-i); anti-PMP70 (mouse monoclonal, IF, Fig 5j); anti-Rab5 (rabbit monoclonal, WB, Fig S8a); anti-Rab11 (mouse monoclonal, WB, Fig S8a); anti-XBP1 (mouse monoclonal, IF, Fig S3f-g, thapsigargin induction); anti-ATL2 (rabbit polyclonal, WB, Fig S11a, CRISPR-KO); anti-ATL3 (rabbit polyclonal, Proteintech, WB, Fig S8a), anti-ATL3 (rabbit polyclonal, ThermoFisher, WB, Fig S11a, CRISPR-KO); anti-BiP (mouse monoclonal, WB, Fig S3e, ER stress induction) ; anti-HA (rat monoclonal 3F10, HRP-conjugated, WB, Fig 2f, S6e).

Eukaryotic cell lines

Policy information about cell lines and Sex and Gender in Research

Cell line source(s)

The following cell lines were commercially obtained: U2OS (ATCC # HTB-96, human XX), SaOS2 (ATCC # HTB-85, human XX derived), Bewo (ATCC # CCL-98, human XY derived); SKN-SH (ATCC # HTB-11, human XX derived), 293FT (ThermoFisher #R70007, human XX derived), Expi293F (ThermoFisher #A14527, human XX derived), and Flp-In 293 (ThermoFisher #R75007, human XX derived). Hela cells (human XX derived) were a gift from Bob Kingston. Phoenix 293 Ampho cells (human XX derived) were a gift from Joseph Brewer. All other cell lines in this study were derived from the above.

Authentication

All commercially purchased parental cell lines were authenticated by the manufacturer using STR profiling. Transgenic and CRISPR-KO cell lines derived from these parental cells were not further authenticated. Hela and Phoenix 293 Ampho cells were not authenticated.

Mycoplasma contamination

All commercially purchased parental cell lines tested negative for mycoplasma contamination by the manufacturer. All cell lines subsequently tested negative for mycoplasma contamination.

Commonly misidentified lines
(See ICLAC register)

No commonly misidentified cell lines in the ICLAC register were used in this study.

Plants

Seed stocks	n/a
Novel plant genotypes	n/a
Authentication	n/a
